# Global transcriptional response of *Caulobacter crescentus* to iron availability

**DOI:** 10.1186/1471-2164-14-549

**Published:** 2013-08-13

**Authors:** José F da Silva Neto, Rogério F Lourenço, Marilis V Marques

**Affiliations:** 1 Departamento de Microbiologia, Instituto de Ciências Biomédicas, Universidade de São Paulo, Av. Prof. Lineu Prestes 1374, 05508-000 São Paulo, SP, Brazil; 2 Departamento de Bioquímica, Instituto de Química, Universidade de São Paulo, Av. Prof. Lineu Prestes 748, 05508-000 São Paulo, SP, Brazil; 3 Present address: Departamento de Biologia Celular e Molecular e Bioagentes Patogênicos, Faculdade de Medicina de Ribeirão Preto, Universidade de São Paulo, Av. Bandeirantes 3900, 14049-900 Ribeirão Preto, SP, Brazil

**Keywords:** *Caulobacter crescentus*, Iron stimulon, Fur regulon, Transcriptome, Iron homeostasis, TonB-dependent receptor

## Abstract

**Background:**

In the alpha subclass of proteobacteria iron homeostasis is controlled by diverse iron responsive regulators. *Caulobacter crescentus*, an important freshwater *α*-proteobacterium, uses the ferric uptake repressor (Fur) for such purpose. However, the impact of the iron availability on the *C. crescentus* transcriptome and an overall perspective of the regulatory networks involved remain unknown.

**Results:**

In this work we report the identification of iron-responsive and Fur-regulated genes in *C. crescentus* using microarray-based global transcriptional analyses. We identified 42 genes that were strongly upregulated both by mutation of *fur* and by iron limitation condition. Among them, there are genes involved in iron uptake (four TonB-dependent receptor gene clusters, and *feoAB*), riboflavin biosynthesis and genes encoding hypothetical proteins. Most of these genes are associated with predicted Fur binding sites, implicating them as direct targets of Fur-mediated repression. These data were validated by β-galactosidase and EMSA assays for two operons encoding putative transporters. The role of Fur as a positive regulator is also evident, given that 27 genes were downregulated both by mutation of *fur* and under low-iron condition. As expected, this group includes many genes involved in energy metabolism, mostly iron-using enzymes. Surprisingly, included in this group are also TonB-dependent receptors genes and the genes *fixK*, *fixT* and *ftrB* encoding an oxygen signaling network required for growth during hypoxia. Bioinformatics analyses suggest that positive regulation by Fur is mainly indirect. In addition to the Fur modulon, iron limitation altered expression of 113 more genes, including induction of genes involved in Fe-S cluster assembly, oxidative stress and heat shock response, as well as repression of genes implicated in amino acid metabolism, chemotaxis and motility.

**Conclusions:**

Using a global transcriptional approach, we determined the *C. crescentus* iron stimulon. Many but not all of iron responsive genes were directly or indirectly controlled by Fur. The iron limitation stimulon overlaps with other regulatory systems, such as the RpoH and FixK regulons. Altogether, our results showed that adaptation of *C. crescentus* to iron limitation not only involves increasing the transcription of iron-acquisition systems and decreasing the production of iron-using proteins, but also includes novel genes and regulatory mechanisms.

## Background

Iron is an essential micronutrient required for almost all organisms, functioning as a cofactor for proteins that are involved in a number of fundamental metabolic and enzymatic functions. Despite its high abundance, iron is a limiting nutrient in most biological systems due to its poor solubility under physiological conditions or because it is tightly sequestered by high-affinity proteins, such as transferrin and lactoferrin in eukaryotic hosts [1,2]. On the other hand, high iron levels can generate toxic hydroxyl radicals by the Fenton reaction [3]. Thus, organisms have evolved multiple strategies to maintain accurate control over intracellular iron levels.

In most bacteria, iron homeostasis is mediated by Fur (ferric uptake regulator), an iron-sensing repressor protein, that controls the expression of genes involved in iron uptake, storage and usage. Under iron sufficiency, Fe^2+^-Fur (holo-Fur) binds at operator sites (Fur boxes) in the promoters of multiple iron-responsive genes, and represses their transcription [4]. In a few bacterial species, Fur seems to have a broader scope of regulation, acting also as a direct transcriptional activator [5-7] or as an apo-regulator (apo-Fur) [8,9]. However, the most common Fur-mediated activation mechanism occurs indirectly via small regulatory RNAs (sRNA), such as RyhB in *Escherichia coli*[10], PrrF1 and PrrF2 in *Pseudomonas aeruginosa*[11], NrrF in *Neisseria meningitidis*[12] and FsrA in *Bacillus subtilis*[13]. In all these cases, the sRNAs inhibit the production of non-essential iron-using proteins under iron limitation, allowing relocation of the intracellular iron for essential proteins [14].

The Fur protein is the most widely found and best-studied iron-responsive regulator in bacteria from diverse taxonomic groups, such as subdivisions γ, β, δ and ϵ of proteobacteria and bacilli [4]. However, in α-proteobacteria iron regulation is still little studied and appears to be mediated by regulators different from Fur. Direct experimental data, available mostly to Rhizobiales, indicate that RirA and Irr are the master regulators of iron homeostasis while a Fur-like protein, named Mur, regulates only a manganese transporter [15,16]. It has been suggested, based on bioinformatics and phylogenetic analyses, that RirA and Irr emerged as the main iron regulators in the common ancestor of the Rhizobiales and Rhodobacterales, whereas in more basal lineages of α-proteobacteria (Caulobacterales, Rhodospirillales and Sphingomonadales), Fur remained as the global iron regulator [17]. This *in silico* prediction was recently confirmed by experimental data for at least two α-proteobacteria, *Caulobacter crescentus*[6] and the magnetotactic bacterium *Magnetospirillum gryphiswaldense*[18,19].

We have previously demonstrated, using an *in silico* approach combined with experimental data, that Fur controls iron homeostasis in *C. crescentus* by regulating many iron-responsive genes, and protect this freshwater oligotrophic bacterium from oxidative stress [6]. However, the response of *C. crescentus* to iron limitation and a comprehensive investigation of its Fur regulon remain to be determined on a global scale. In this work, we performed DNA microarray analysis to determine the transcriptional response of *C. crescentus* to iron availability, using wild-type cells growing under iron-replete *versus* iron-limiting conditions. We also used transcriptional profiling, comparing wild-type *versus fur*-mutant strains, to find novel members of the *C. crescentus* Fur regulon.

## Results and discussion

### Effect of iron and Fur on the *C. crescentus* transcriptome

**Figure 1:**
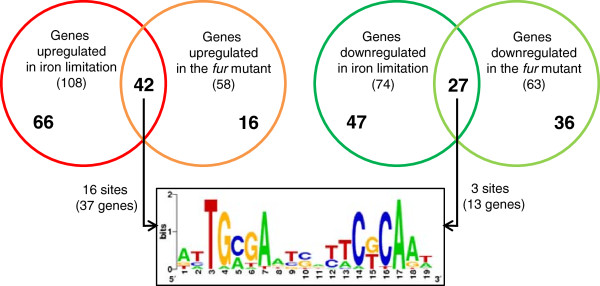
**Overview of iron-responsive and Fur-regulated genes in *C. crescentus *identified by microarray analyses.** The Venn diagrams were constructed using the number of up- and down-regulated genes from experiments comparing wild type cells exposed to iron-limiting *versus* iron-replete conditions or comparing *fur* mutant strain *versus* wild type strain both in iron-replete condition. The complete set of the genes belonging to each group is listed in Tables 1, 2, 3, 4 and Additional file 1: Table S1. The upstream region of these genes (−200 to +50 bp relative to the start codon) were searched for sequence motifs using the MEME tool. A 19-pb palindromic motif, corresponding to the Fur binding site, was exclusively found in the group of genes regulated by both iron and Fur.

Whole genome transcriptional profiling using DNA microarrays were performed to identify iron-responsive and Fur-regulated genes in *C. crescentus*. Two sets of microarray experiments were conducted in duplicate using RNA samples prepared from two independent biological cultures. First, to define the *C. crescentus* iron limitation stimulon, we compared the transcriptome of wild-type cells treated with 100 μM FeSO_4_ (iron sufficiency) with that of wild-type cells treated with 100 μM 2,2-dipyridyl for 2 hours (iron limitation), an experimental condition previously established to study gene expression of iron-responsive genes in *C. crescentus*[6]. Iron limitation altered expression of 182 genes of which 108 were upregulated and 74 downregulated (Figure 1). Second, to identify Fur-regulated genes, we compared the transcriptome of wild-type cells with that of a *fur* mutant both cultivated in iron sufficiency. The expression of 121 genes was found to be significantly changed by the *fur* mutation (58 upregulated genes and 63 downregulated genes) (Figure 1).

The up- and down-regulated genes, identified in these two microarray experiments, were compared to identify genes regulated by both iron limitation and *fur* mutation or genes affected by only one of these conditions (Figure 1). We found 42 genes upregulated both under iron limitation and in the *fur* mutant (Fe^2+^-Fur repressed genes) and 27 genes that were found to be downregulated on both these conditions (Fe^2+^-Fur activated genes), indicating that Fur has a major role on controlling expression of iron-responsive genes in *C. crescentus*. We also found many genes regulated exclusively in response to iron limitation, namely 66 upregulated genes and 47 downregulated genes, suggesting that the *C. crescentus* iron limitation stimulon is controlled by additional regulatory mechanisms.

Lastly, a group of genes showed differential expression in the *fur* mutant (16 up- and 36 downregulated genes) independent of iron availability (Figure 1; Additional file 1: Table S1). We were unable to determine whether these transcriptional changes are secondary effects or are mediated directly by Fur in an iron-independent manner. Nevertheless, the most upregulated genes in the *fur* mutant are the genes involved in transport (CC0859-60-61) and catabolism (CC1296, CC1298, CC1299 and CC1302) of *myo*-inositol in *C. crescentus*, belonging to the IolR regulon [20]. As expected, the level of *fur* mRNA (CC0057) was severely reduced in the *fur* mutant (7.4 fold). Interestingly, the *sodB* gene (CC3557) encoding an iron/manganese superoxide dismutase was 2.2-fold downregulated in the *fur* mutant (Additional file 1: Table S1), although its iron-dependent regulation verified in other bacteria [21] was not observed in our microarrays.

### The repertoire of iron-responsive and Fur-regulated genes in *C. crescentus*

**Figure 2:**
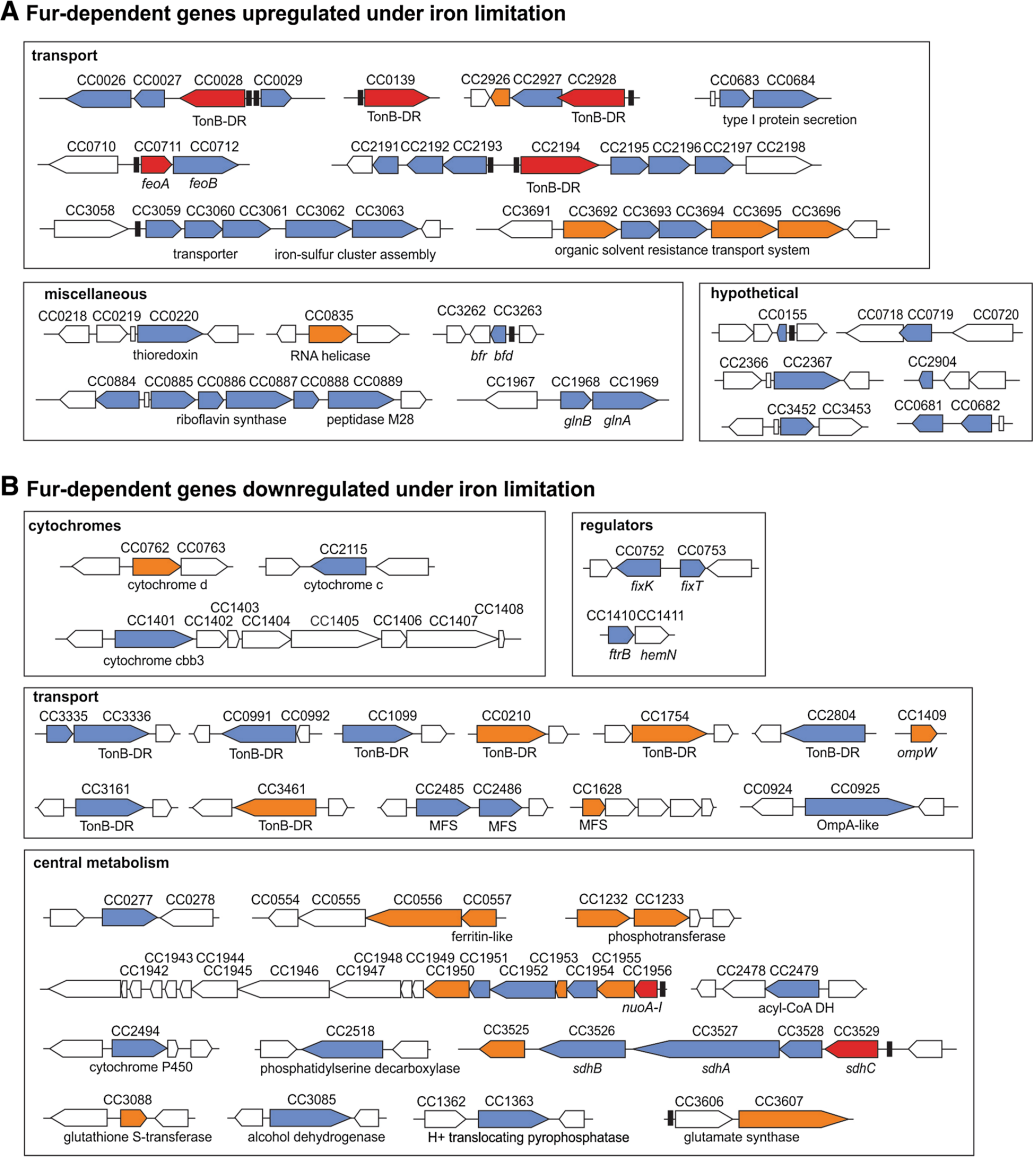
**Genomic organization of the *C. crescentus *Fur regulon.** The chromosomal clusters of the iron-responsive and Fur-regulated genes are organized in functional categories grouped in separate panels. Genes are also grouped as upregulated **(A)** or downregulated **(B)** under both iron limitation and *fur* mutation. The arrows indicate each open reading frame and their orientation on the chromosome. Differentially expressed genes are indicated in blue. Genes that have been experimentally shown to be directly regulated by Fur [6] are indicated in red. Selected genes that were either iron or Fur regulated (Tables 3, 4 and Additional file 1: Table S1) and showed expression change very close to our cutoff criterion on the other condition are shown in orange. Vertical blocks indicate the location of the Fur binding sites detected by the MEME search described in Figure 1, where sites predicted *in silico* are white and experimentally validated by EMSA are black [6].

The genes regulated by both iron limitation and Fur are those showed in Tables 1 and 2 (in Figure 2A and 2B, genes indicated in blue). In addition to those genes, we observed that expression of some genes assumed as significantly up or downregulated under only one of the tested conditions changed to levels very close to our cutoff criterion (twofold change) in the other condition (Tables 3, 4 and Additional file 1: Table S1 and in Figure 2, genes indicated in orange). Thus, it is probable that these genes are also responsive to both iron levels and Fur, especially if one considers that most of them are in putative operons with genes whose expression was significantly changed under both iron limitation and *fur* mutation. Therefore, these genes were discussed here as part of the repertory of iron-responsive and Fur-regulated genes.

**Table 1:** **Genes upregulated under iron-limiting condition and in the *fur *mutant**

**Gene CB15**	**Gene NA1000**	**Predicted function**^**a**^	**Fold change**^**b**^	
			**WT DP/WT Fe**	**∆*fur *Fe/WT Fe**
**Transport**				
CC_0026	CCNA_00026	PAS-family sensor histidine kinase (heme)	4.70	5.95
CC_0027	CCNA_00027	PKHD-type hydroxylase (FeII)	15.65	26.39
CC_0028^c^	CCNA_00028	TonB-dependent receptor	28.27	55.08
CC_0029	CCNA_00029	Lysine exporter protein	2.18	2.03
CC_0139	CCNA_00138	TonB-dependent receptor	20.21	33.27
CC_0683	CCNA_00719	Type I secretion adaptor protein hlyD	2.42	2.81
CC_0684	CCNA_00720	Type I protein secretion ATP-binding protein	2.25	2.80
CC_0711	CCNA_00748	Ferrous iron transport protein A	9.10	9.04
CC_0712	CCNA_00749	Ferrous iron transport protein B	6.13	5.96
CC_2191	CCNA_02272	Hypothetical protein	4.49	7.10
CC_2192	CCNA_02273	Glutathione peroxidase (DUF3297)	6.27	9.09
CC_2193	CCNA_02274/75	EF hand protein/hypothetical protein (DUF4198)	64.82	167.73
CC_2194	CCNA_02277	Hemin receptor (TonB-dependent receptor)	17.90	25.29
CC_2195	CCNA_02278	Putative membrane-associated alkaline phosphatase	4.49	7.27
CC_2196	CCNA_02279	Disulfide bond formation protein B	2.51	3.17
CC_2197	CCNA_02280	Ubiquinone biosynthesis protein COQ7 (Iron)	2.52	3.23
CC_2927	CCNA_03022	Transporter	27.09	34.54
CC_2928	CCNA_03023	TonB-dependent receptor	15.36	22.72
CC_3059	CCNA_03155	Transporter	23.57	22.29
CC_3060	CCNA_03156	Putative periplasmic protein (DUF2271)	24.53	32.44
CC_3061	CCNA_03157	Putative membrane spanning protein (DUF4198)	44.13	51.11
CC_3062	CCNA_03158	Iron-sulfur cluster assembly/repair protein ApbE	17.44	24.66
CC_3063	CCNA_03159	Sulfite reductase (NADPH) flavoprotein (Heme)	12.25	16.47
CC_3693	CCNA_03807	Organic solvent resistance transport system Ttg2D protein	6.48	2.50
CC_3694	CCNA_03808	Organic solvent resistance transport system Ttg2C protein	5.62	2.19
**Riboflavin biosynthesis**				
CC_0885	CCNA_00929	Diaminohydroxyphosphoribosylaminopyrimidine deaminase	10.83	4.99
CC_0886	CCNA_00930	Riboflavin synthase alpha chain	8.01	3.57
CC_0887	CCNA_00931	3,4-dihydroxy-2-butanone-4-phosphate synthase	12.18	3.70
CC_0888	CCNA_00932	6,7-dimethyl-8-ribityllumazine synthase	13.72	4.33
CC_0889	CCNA_00933	Putative peptidase	5.75	3.08
**Miscellaneous**				
CC_0220	CCNA_00220	Thiol-disulfide isomerase and thioredoxin	3.80	3.31
CC_0884	CCNA_00928	Transcriptional regulator, GntR family	5.16	2.45
CC_1968	CCNA_02046	Nitrogen regulatory protein P-II GlnB	2.87	3.45
CC_1969	CCNA_02047	Glutamine synthetase GlnA	2.32	2.33
CC_3263	CCNA_03372	Bacterioferritin-associated ferredoxin (Fe-S cluster)	56.96	40.04
**Hypothetical**				
CC_0155	CCNA_00154	Hypothetical protein DUF2061 (predicted membrane)	13.07	5.99
CC_0681	unannotated	Hypothetical protein	7.97	3.77
CC_0682	unannotated	Hypothetical protein	10.03	3.88
CC_0719	CCNA_00756	Hypothetical protein	9.81	2.89
CC_2367	CCNA_02452	Hypothetical protein	23.64	26.74
CC_2904	CCNA_02998	Hypothetical protein	9.64	13.35
CC_3452	CCNA_03566	Hypothetical protein	2.52	2.51

^a^ The terms in parenthesis are Pfam domains found in hypothetical proteins or metals predicted to bind the proteins. Metal cofactors were found by searching the ExPASy and Brenda databases.

^b^ Values are fold changes in the expression levels comparing wild type cells exposed to iron-limiting *versus* iron-replete conditions (WT DP/ WT Fe) or comparing *fur* mutant strain *versus* wild type strain both exposed to iron-replete condition (∆*fur* Fe/WT Fe). The values were obtained as the average of the four last probes for each gene.

^c^ According to previously proposed in earlier work [6], the most probable initiation codon of CC0028 is at position +234 relative to the initiation codon annotated in the genome. Thus, the last four probes designed for CC0028 are not useful to measure its expression. The values showed for this gene correspond to the average of four initial probes of the CC0027 gene, which hybridize within the final portion of CC0028.

**Table 2:** **Genes downregulated under iron-limiting conditions and in the *fur *mutant**

**Gene CB15**	**Gene NA1000**	**Predicted function**	**Fold change**^**a**^	
			**WT DP/WT Fe**	**∆*fur *Fe/WT Fe**
**Transport**				
CC_0925	CCNA_00974	OAR protein precursor (OmpA-like protein)	−8.22	−2.74
CC_0991	CCNA_01042	TonB-dependent receptor	−2.47	−2.27
CC_1099	CCNA_01155	TonB-dependent outer membrane receptor	−2.22	−2.06
CC_2485	CCNA_02570	Transporter (Major Facilitator Superfamily)	−2.49	−3.24
CC_2486	CCNA_02571	Transporter (Major Facilitator Superfamily)	−2.16	−2.24
CC_2804	CCNA_02895	TonB-dependent receptor	−2.41	−2.22
CC_3161	CCNA_03263	TonB-dependent receptor	−2.89	−2.68
CC_3335	reannotated	Hypothetical protein	−4.54	−5.88
CC_3336	CCNA_03444	TonB-dependent receptor	−2.51	−2.38
**Energy Metabolism**				
CC_0277	CCNA_00279	NAD(P)H dehydrogenase (quinone)	−3.11	−4.74
CC_1401	CCNA_01467	Cytochrome cbb3 oxidase subunit I ccoN	−2.76	−6.07
CC_1951	CCNA_02028	NTF2 enzyme family protein	−2.11	−2.23
CC_1952	CCNA_02029	NADH-quinone oxidoreductase chain D	−2.18	−2.23
CC_1954	CCNA_02031	NADH-quinone oxidoreductase chain C	−2.09	−2.16
CC_2115	CCNA_02200	Cytochrome c-family protein	−3.02	−4.61
CC_2494	CCNA_02579	Cytochrome P450 (Heme)	−3.31	−4.43
CC_3526	CCNA_03641	Succinate dehydrogenase iron-sulfur protein (Fe-S cluster)	−2.35	−3.12
CC_3527	CCNA_03642	Succinate dehydrogenase flavoprotein subunit	−2.84	−2.95
CC_3528	CCNA_03643	Succinate dehydrogenase membrane anchor subunit	−3.11	−3.29
CC_3529	CCNA_03644	Succinate dehydrogenase cytochrome B-556 subunit	−2.87	−2.89
**Miscellaneous**				
CC_1363	CCNA_01425	H+ translocating pyrophosphatase	−3.35	−2.78
CC_2479	CCNA_02564	acyl-CoA dehydrogenase, short-chain specific	−2.38	−2.29
CC_2518	CCNA_02603	Phosphatidylserine decarboxylase (DUF1254)	−3.05	−4.79
CC_3085	CCNA_03181	Alcohol dehydrogenase (Zinc or iron)	−4.05	−2.28
**Regulators**				
CC_0752	CCNA_00789	Hypoxia transcriptional regulator FixK	−3.02	−4.08
CC_0753	CCNA_00790	Hypoxia negative feedback regulator FixT	−2.63	−3.65
CC_1410	CCNA_01476	CRP-family transcription regulator FtrB	−6.56	−13.47

^a^ Values are fold changes in the expression levels as described in Table 1. Negative values denote downregulation.

**Table 3:** Genes upregulated exclusively in response to iron limitation

**Gene CB15**	**Gene NA1000**	**Predicted function**	**Fold change**^**a**^
**Amino acid metabolism**			
CC_0013	CCNA_00013	Protein-PII uridylyltransferase GlnD	2.62
CC_0272	CCNA_00273	Peptide deformylase (FeII)	2.81
CC_0977^b^	CCNA_01028	Cytosol aminopeptidase (Zinc or Manganese)	4.62
CC_1612	CCNA_01684	Phenylalanine-4-hydroxylase (Iron)	2.33
CC_2481^b^	CCNA_02566	Membrane alanine aminopeptidase (Zinc)	2.90
CC_2532	CCNA_02615	Homogentisate 1,2-dioxygenase (Iron)	2.38
CC_2533	CCNA_02616	4-hydroxyphenylpyruvate dioxygenase (Iron)	2.49
CC_3686	CCNA_03800	Diaminopimelate epimerase	2.25
**Iron-sulfur cluster assembly/repair**			
CC_0061	CCNA_00059	Oxygen-insensitive NADH nitroreductase	3.31
CC_0062	CCNA_00060	Mitochondrial-type Fe-S cluster assembly protein NFU	4.60
CC_0132^b^	CCNA_00131	Rrf2 family protein	3.30
CC_1857	CCNA_01933	Hypothetical protein	5.66
CC_1858	CCNA_01934	HesB protein family	5.66
CC_1859	CCNA_01935	FeS assembly SUF system protein	5.50
CC_1860	CCNA_01936	Cysteine desulfurase/Selenocysteine lyase	7.58
CC_1861	CCNA_01937	SufD protein	5.90
CC_1862	CCNA_01938	ATP-dependent transporter sufC	7.89
CC_1863	CCNA_01939	ADP-ribosylglycohydrolase	8.30
CC_1864	CCNA_01940	ABC transporter-associated protein sufB	7.81
CC_1865	CCNA_01941	Cysteine desulfhydrase/Selenocysteine lyase	7.09
CC_1866^b^	CCNA_01942	Rrf2 family transcriptional regulator	7.98
**Oxidative stress**			
CC_0141	CCNA_00140	Glutathione synthetase	2.55
CC_0993	CCNA_01045	Conserved hypothetical cytosolic protein (DUF419)	2.84
CC_0994^b^	CCNA_01046	Peptide methionine sulfoxide reductase msrA	3.07
CC_1315	CCNA_01375	Lactoylglutathione lyase	2.97
CC_1316^b^	CCNA_01376	Glutathione S-transferase	3.93
**Heat shock response**			
CC_0685	CCNA_00721	Chaperonin GroEL	2.33
CC_0686^b^	CCNA_00722	Co-chaperonin GroES	2.27
CC_0878	CCNA_00922	ClpB protein	2.71
CC_2258	CCNA_02341	Small heat shock protein	5.50
CC_2467	CCNA_02552	ATP-dependent Clp protease adaptor protein ClpS	2.42
CC_2468	CCNA_02553	ATP-dependent clp protease ATP-binding subunit ClpA	2.63
CC_2509	CCNA_02594	Endopeptidase htpX	8.09
CC_2510^b^	CCNA_02595	Hypothetical protein	8.94
CC_3098^b^	CCNA_03195	RNA polymerase sigma factor RpoH	5.16
CC_3592^b^	CCNA_03706	Small heat shock protein	3.42
CC_3727^b^	CCNA_03843	ATP-dependent endopeptidase hsl proteolytic subunit hslV	3.48
CC_3728	CCNA_03844	ATP-dependent endopeptidase hsl ATP-binding subunit hslU	2.49
**Detoxification/Resistance**			
CC_0321	CCNA_00323	Low-affinity zinc transport protein	2.80
CC_0807	CCNA_00850	Cation/multidrug efflux pump acrB2	4.59
CC_0808	CCNA_00851	Periplasmic multidrug efflux lipoprotein precursor	4.14
CC_3195^b^	CCNA_03299	Outer membrane protein oprM	3.32
CC_3197	CCNA_03301	Cation/multidrug efflux pump acrB	2.70
CC_3443	CCNA_03556	Quaternary ammonium compound-resistance protein	2.34
CC_3681^b^	CCNA_03795	Tellurium resistance protein terB	4.33
**DNA metabolism**			
CC_0260	CCNA_00261	Ribonucleoside-diphosphate reductase beta chain (Iron)	2.79
CC_2229	CCNA_02312	SLA2 protein (TraB family)	2.64
CC_2590	CCNA_02673	Excinuclease ABC subunit A	2.22
CC_3492	CCNA_03607	Ribonucleoside-diphosphate reductase alpha chain (Iron)	3.51
**Miscellaneous**			
CC_0653	CCNA_00690	CarD-like transcriptional regulator	2.14
CC_0679	CCNA_00718	Abortive infection protein	2.52
CC_0827	CCNA_00870	Putative cytosolic protein (DUF1178)	2.77
CC_0883	CCNA_00927	Hypothetical protein	3.65
CC_2018	CCNA_02097	Periplasmic glucan glucosyltransferase	2.11
CC_2129	CCNA_02213	NADH dehydrogenase (Fe-S cluster)	3.60
CC_2506	CCNA_02592	Thioesterase	2.48
CC_2653^b^	CCNA_02736	Nitroreductase family	3.37
CC_2659	CCNA_02742	Oxalate/formate antiporter (MSF transporter)	3.11
CC_2926^c^	CCNA_03021	Hypothetical protein	4.14
CC_3002	CCNA_03097	Aldo/keto reductase family protein	4.69
CC_3019	CCNA_03113	Membrane-associated phospholipid phosphatase	3.48
CC_3385^b^	CCNA_03496	Putative cytosolic protein (DUF328)	2.96
CC_3406	CCNA_03517	Cytochrome c oxidase polypeptide I coxA	2.32
CC_3692^c^	CCNA_03806	Outer membrane lipoprotein	11.60
CC_3695^c^	CCNA_03809	Organic solvent resistance transport system permease	4.68
CC_3696^c^	CCNA_03810	Organic solvent resistance transport system ATP-binding protein	4.81

^a^ Values are fold changes in the expression levels comparing wild type cells exposed to iron-limiting *versus* iron-replete conditions (WT DP/ WT Fe).

^b^ Promoters of these genes have a predicted RpoH-binding motif identified in McGrath *et al*., (2007) [39].

^c^These genes are probably also upregulated in the *fur* mutant since their expression changes were very close to our cutoff criterion.

**Table 4:** Genes downregulated exclusively in response to iron limitation

**Gene CB15**	**Gene NA1000**	**Predicted function**	**Fold change**^**a**^
**Amino acid metabolism**			
CC_0049	CCNA_00047	tRNA m7-G46 methyltransferase	−3.12
CC_0050	CCNA_00048	S-adenosylmethionine synthetase	−3.05
CC_0167	CCNA_00166	Hypothetical protein (transglutaminase-like cysteine proteinase)	−2.22
CC_0257	CCNA_00257	Adenosylhomocysteinase	−3.12
CC_0482	CCNA_00515	Cobalamin-independent methionine synthase (Zinc)	−2.49
CC_0984	CCNA_01035	Gamma-glutamyltranspeptidase	−2.68
CC_1048	CCNA_01100	Acylamino-acid-releasing enzyme	−2.73
CC_2137	CCNA_02221	Methionine synthase I metH (Zinc)	−2.52
CC_2138	CCNA_02222	5-methyltetrahydrofolate	−2.72
CC_2139	CCNA_02223	Beta-lactamase, type II (Zinc)	−2.83
CC_2140	CCNA_02224	Methylenetetrahydrofolate reductase	−2.39
CC_2840	CCNA_02933	Aminopeptidase	−2.14
CC_3044	CCNA_03139	Dihydroxy-acid dehydratase (Fe-S cluster)	−3.35
CC_3246	CCNA_03355	Acylamino-acid-releasing enzyme	−2.20
CC_3606	CCNA_03721	Glutamate synthase (NADPH) small chain	−2.30
CC_3607^b^	CCNA_03722	Glutamate synthase (NADPH) large chain (Fe-S cluster)	−2.51
**Chemotaxis and motility**			
CC_0430	CCNA_00439	Methyl-accepting chemotaxis protein	−2.97
CC_0431	CCNA_00440	CheX protein	−2.50
CC_0432	CCNA_00441	Chemotaxis receiver domain protein cheYI	−2.31
CC_0433	CCNA_00442	Chemotaxis histidine kinase protein cheAI	−2.11
CC_0901	CCNA_00946	Basal-body rod modification protein FlgD	−2.38
CC_0902	CCNA_00947	Flagellar hook protein FlgE	−2.23
CC_1399	CCNA_01465	Methyl-accepting chemotaxis protein	−2.22
CC_1456	CCNA_01523	Acetyltransferase flmH	−2.63
CC_2846	CCNA_02939	Conserved hypothetical protein	−5.24
CC_2847	CCNA_02940	Methyl-accepting chemotaxis protein	−3.44
**Energy Metabolism**			
CC_1942	CCNA_02020	NADH-quinone oxidoreductase chain I (Fe-S cluster)	−2.12
CC_1943	Unannotated	Hypothetical protein	−2.20
CC_1944	CCNA_02021	Hypothetical protein	−2.26
CC_1946	CCNA_02023	NADH-quinone oxidoreductase chain G (Fe-S cluster)	−2.05
CC_1953^b^	CCNA_02030	Hypothetical protein	−2.19
CC_3525^b^	CCNA_03640	Ferredoxin reductase subunit (Fe-S cluster)	−2.30
CC_3659	CCNA_03774	Citrate lyase beta chain/citryl-CoA lyase subunit	−2.00
CC_3667	CCNA_03781	Aconitate hydratase (Fe-S cluster)	−2.30
**Miscellaneous**			
CC_0566	CCNA_00601	MoxR-like ATPase	−2.04
CC_1409^b^	CCNA_01475	OmpW family outer membrane protein	−3.21
CC_1754^b^	CCNA_01830	TonB-dependent receptor	−2.07
CC_2389	CCNA_02472	Cobalt-zinc-cadmium resistance protein czcB	−2.42
CC_3081	CCNA_03177	Methylmalonyl-CoA mutase MeaA-like protein	−2.55
CC_3127^b^	CCNA_03227	TonB-dependent receptor	−2.41
CC_3413	CCNA_03524	Di-/tripeptide transporter (Major Facilitator Superfamily)	−2.48
CC_3461^b^	CCNA_03574	TonB-dependent receptor	−2.70
**Hypothetical**			
CC_0600	CCNA_00636	Hypothetical protein	−2.16
CC_1068	CCNA_01121	Conserved hypothetical protein	−2.40
CC_1102	CCNA_01158	Hypothetical protein	−2.47
CC_2745^b^	CCNA_02831	Conserved hypothetical protein (DUF2272)	−3.14
CC_3412	CCNA_03523	Hypothetical protein (Acetyltransferase (GNAT) family)	−2.18

^a^ Values are fold changes in the expression levels as described in Table 3. Negative values denote downregulation.

^b^These genes are probably also downregulated in the *fur* mutant since their expression changes were very close to our cutoff criterion.

The genes upregulated by both iron limitation and *fur* mutation (Fe^2+^-Fur repressed genes) were grouped into functional categories and according to their transcriptional organization in the chromosome (Table 1; Figure 2A). Many of these genes are organized in large clusters that contain at least one gene predicted to be involved in transport, implicating them in iron-acquisition associated functions (Figure 2A). These include four gene clusters containing TonB-dependent receptors, which are outer membrane proteins probably involved in Fe^3+^-siderophore acquisition (CC0028-27-26, CC0139, CC2194-95-96-97 and CC2928-27-26), the operon encoding the ferrous iron transporter FeoAB (CC0711-12) as well as two gene clusters encoding predicted ABC transporters (CC3692-93-94-95-96 and CC0683-84) and two gene clusters encoding hypothetical proteins that are putative transporters (CC2193-92-91 and CC3059-60-61-62-63) (these last two operons are discussed below). Although none of these putative transporters have been characterized yet, their high derepression by both iron limitation and *fur* mutation (Table 1) indicates that they could play a major role in the adaptation of *C. crescentus* to low-iron conditions. Unexpectedly, it has been shown, using hyper-saturated transposon mutagenesis, that *feoAB* is an essential operon in *C. crescentus* even for growth on rich media (iron sufficiency) [22], highlighting the vital role of iron acquisition in this bacterium.

In addition to these putative iron acquisition systems, a riboflavin biosynthesis operon (CC0885-86-87-88-89) as well as the *bfd* gene (CC3263) encoding a ferredoxin associated with bacterioferritin were upregulated by both iron limitation and *fur* mutation (Table 1; Figure 2A). It has been reported for *Helicobacter pylori* and *Campylobacter jejuni* that the production of riboflavin is also regulated by iron and Fur and secreted riboflavin has a role in Fe^3+^ reduction and hence in iron acquisition [23,24]. Genes involved in oxidative stress response (CC0220), RNA processing (CC0835), transcriptional regulation (CC0884) and ammonia assimilation (CC1968-69) were also Fe^2+^-Fur repressed. A tight connection between iron homeostasis and nitrogen metabolism has been reported for the nitrogen-fixing cyanobacterium *Anabaena sp*. [25].

Finally, seven genes encoding hypothetical proteins were also upregulated by both iron limitation and *fur* mutation, of which two genes are of particular interest (CC0681 and CC0682). A previous report, based on tiled microarray analysis, suggested the existence of two candidate small regulatory RNAs (sRNAs) located in the intergenic regions between CC0680-CC0681 and C00681-CC0682, but attempts to validate these sRNAs by Northern blot allowed the detection of only a large transcript comprising all this region [26]. Considering that the putative operon CC0682-sRNA1-CC0681-sRNA2 was found to be Fe^2+^-Fur repressed in our microarray analyses (Table 1, Figure 2A) we are tempted to speculate that it could be processed under iron limitation, generating two sRNAs and two mRNAs translated to small proteins. These components could mediate the iron sparing response in *C. crescentus*, similarly to what was observed in *Bacillus subtilis* in which a sRNA (FsrA) and three small basic proteins (FbpA, FbpB e FbpC) act in conjunction to repress the expression of iron-rich proteins [13].

Additionally to these Fe^2+^-Fur repressed genes, our microarray analyses allowed us to identify the genes positively regulated by Fe^2+^-Fur, in other words, the genes that were downregulated by both iron limitation and *fur* mutation (Table 2; Figure 2B). As expected, many of these genes encode iron-containing enzymes. These included succinate dehydrogenase (*sdh* operon, CC3529-28-27-26-25), NADH ubiquinone oxidoreductase (*nuo* operon, CC1956-55-54-53-52-51-50), cytochromes (CC0762, CC1401 and CC2115), cytochrome P450 enzyme (CC2494), glutamate synthase (CC3607), a hypothetical protein predicted as catalase and a hypothetical protein with a ferritin-like domain (CC0556-57). This mechanism of repressing iron-rich enzymes to prioritize iron usage when this metal is scarce, sometimes referred as iron sparing response, has been described in many bacteria, such as *E. coli*[10,21,27], *P. aeruginosa*[11] and *B. subtilis*[13,28].

Unexpectedly, a large number of genes encoding proteins involved in transport were also downregulated by both iron limitation and *fur* mutation (Table 2; Figure 2B). Among these, there are transporters belonging to the major facilitator superfamily (MFS) (CC1628, CC2485-86), porins (CC0925 and CC1409) and many TonB-dependent receptors. At least six of these genes (CC3336, CC3161, CC3461, CC0991, CC2804 and CC2485) are also highly induced by carbon limitation [29] and are positively regulated by CfrA, a sRNA that regulates adaptation to carbon starvation in *C. crescentus*[30]. Although the reason for these genes to be repressed by iron limitation and induced by carbon starvation is still not clear, it is reasonable to suppose that these TonB-dependent receptors are required for uptake of carbohydrates instead of Fe^3+^-siderophore complexes, since it has recently been shown that novel substrates, such as nickel and different carbohydrates, are transported via TonB-dependent receptors [31].

Importantly, three genes (*fixK*, *fixT* and *ftrB*) encoding regulatory proteins that specify an oxygen signaling network required for *C. crescentus* growth under hypoxia [32] were found to be downregulated by both iron limitation and *fur* mutation (Table 2; Figure 2B). The *C. crescentus* Fix signaling system consists of the sensor histidine kinase FixL (a heme-binding oxygen sensor), its cognate response regulator FixJ, the transcriptional regulator FixK, and the kinase inhibitor FixT (the core FixLJ–FixK–FixT), besides the downstream regulators FtrA and FtrB [32]. Consistent with downregulation of *fixK*, many hypoxia-dependent FixK-activated genes containing a FixK binding site [32], were also downregulated by both iron limitation and *fur* mutation, including CC1409 (*ompW*), CC1410 (*ftrB*), CC0762 (*cydA*), CC1401 (*ccoN*), CC0753 (*fixT*), CC2115 and CC0277 (Table 2; Figure 2B). Therefore, the FixK-dependent hypoxia stress response seems to be positively regulated by Fe^2+^-Fur under iron sufficiency and repressed in iron limitation condition, similarly to what was described for the anaerobic regulator Fnr in *E. coli*[21] and *Salmonella enterica serovar Typhimurium*[33]. The regulatory link between oxygen and iron availability could be mediated by the histidine kinase FixL that senses oxygen through its heme-containing amino-terminal PAS domain [32].

To further discriminate whether regulation by Fur was direct or indirect, we conducted *in silico* searches in the upstream region of all up- and down-regulated genes identified in the microarray experiments (Figure 1). MEME-based analyses, including all genes together or each group of genes separately, identified a motif very similar to the Fur binding site previously detected in *C. crescentus*[6]. These Fur binding sites were detected only for genes regulated by both iron and Fur (Figure 1). As indicated in Figure 2, sixteen Fur binding sites were identified in the group of the genes upregulated by both iron limitation and *fur* mutation, indicating that most of these genes (37 out of 47 genes) are direct target for Fur-mediated repression. In contrast, only three Fur binding sites were detected in the group of the genes downregulated by both iron limitation and *fur* mutation, suggesting that Fur indirectly mediates positive regulation of many genes, in addition to the direct positive regulation previously demonstrated [6].

### Fur-independent regulation of *C. crescentus* iron-responsive genes

In addition to the Fur modulon iron limitation also affected the *C. crescentus* transcriptome in a Fur-independent manner, given that 66 genes were upregulated (Table 3) and 47 genes were downregulated (Table 4) during growth in iron-limitation condition that were not affected by the *fur* mutation (Figure 1).

Among the genes strongly upregulated exclusively in response to iron limitation there is a large gene cluster (CC1866-65-64-63-62-61-60-59-58-57), which encodes the transcriptional repressor IscR (CC1866) and enzymes of the Suf system of Fe-S cluster biogenesis (Table 3). *E. coli* possesses two operons implicated in Fe-S cluster assembly, *iscRSUA-hscBA-fdx*, encoding the housekeeping Fe-S cluster biogenesis pathway and *sufABCDSE*, which synthesize Fe-S clusters under iron limitation or oxidative stress conditions [34,35), whereas *C. crescentus* appears to have only one operon that contains a combination of *isc* (CC1866-65, *iscRS*) and *suf* (CC1864-62-61-60, *sufBCDS*) genes. In *E. coli* both *isc* and *suf* operons are induced by iron limitation and oxidative stress, but while the *isc* genes are regulated by IscR, the *suf* genes are under control of OxyR and Fur [21,34-36]. In *C. crescentus* upregulation of this large operon by iron limitation is Fur-independent and we postulate that it could be mediated by IscR via an IscR binding site previously predicted upstream of the CC1866 gene [17]. Because IscR senses damage to the Fe-S clusters of the cell, it is possible that iron limitation is generating some kind of stress in *C. crescentus* which is able to damage Fe-S clusters.

In agreement with this assumption, many of the genes upregulated exclusively by iron limitation are related to various stress responses (Table 3) and were found to be induced when *C. crescentus* was submitted to heavy metal stress [37]. Among the genes induced by both iron limitation and heavy metal stress (mainly cadmium stress), there are those related to oxidative stress defense (CC0141, CC0994, CC1316), detoxification efflux pumps (CC3195, CC3197), DNA repair (CC2590) and nucleotide biosynthesis (CC0260, CC3492) (Table 3). Interestingly, 12 heat shock genes, encoding chaperones, proteases and small heat shock proteins, were also upregulated by iron limitation, as well as some genes encoding peptidases containing metals as cofactors (Table 3), what is consistent with previous observations in *Shewanella oneidensis*[38]. Induction of these genes might be directly mediated by the heat shock sigma factor RpoH (σ^32^), for the reason that the own *rpoH* gene (CC3098) is upregulated in iron limitation (Table 3). Moreover, a predicted σ^32^-binding motif (m_6 motif), which has been identified upstream of cadmium-induced genes [39], was found here upstream of nearly half (15 sites upstream of 30 genes/operons) of the 63 genes upregulated in iron limitation (Table 3), indicating induction of the RpoH regulon by iron limitation. The *C. crescentus rpoH* gene is transcribed from two promoters, a σ^70^-dependent P1 promoter and a heat shock autoregulated σ^32^-dependent P2 promoter [40]. It remains to be determined how these different signals (cadmium stress and iron limitation) could increase transcription of *rpoH* in *C. crescentus*, activating its regulon.

When the genes downregulated exclusively in iron limitation are grouped into functional categories, the most prominent groups of genes are involved in amino acid metabolism, chemotaxis and motility, and energy metabolism (Table 4). Among the enzymes of amino acid biosynthesis pathways repressed by iron limitation there are many involved in methionine biosynthesis, such as methionine synthases (CC0482, CC2137, CC2138), adenosylmethionine synthtase (CC0050), S-adenosyl-L-homocysteine hydrolase (CC0257) and methylenetetrahydrofolate reductase (CC2140), which is required to produce 5-methyltetrahydrofolate as methyl-group donor for methionine synthesis. Pathways of protein catabolism were also repressed by iron limitation as revealed by downregulation of many genes encoding peptidases (CC0167, CC0984, CC1048, CC2480 and CC3246) (Table 4). Furthermore, some genes for flagella assembly (CC0901-02, CC1456) and chemotaxis (CC0430-31-32-33, CC1399 and CC2847) were downregulated in iron limitation. Repression of motility and chemotaxis genes by iron limitation has been described in *Sinorhizobium meliloti*[41] and *Acinetobacter baumannii*[42]. Finally, some known Fe^2+^-Fur activated genes [6,13] were downregulated in iron limitation, but not in the *fur* mutant in this work. Of these, there are genes encoding the Fe-S clusters-containing enzymes aconitate hydratase (CC3667), NADH ubiquinone oxidoreductase (*nuo* genes CC1946, CC1944-43-42), glutamate synthase (CC3606) and dihydroxy-acid dehydratase (CC3044) (Table 4). In some cases, at least part of the operons (*nuo* and CC3607) was downregulated by both iron limitation and *fur* mutation (Figure 2B). A possible explanation is that the Fe^2+^-Fur activated genes showed modest differential expression (approximately 2 fold) (Table 2), thus small experimental fluctuations could exclude some genes based on our cutoff criteria for differential expression in the microarray analyses.

Comparing our microarray data with other large-scale transcriptomic studies performed under iron-limiting condition in bacteria from diverse taxonomic groups [21,28,38,41,42], we observed that, in spite of the multiplicity of regulatory mechanisms, the core of iron-regulated genes is extremely conserved, including mainly those related to transport, use and storage of this metal. Some responses seems to be confined to few bacteria, such as upregulation of the heat shock response, also described in *S. oneidensis*[38] and downregulation of chemotaxis and motility, observed in *S. meliloti*[41] and *A. baumannii*[42]. However, our study expands the range of genes involved in iron homeostasis when we consider physiological processes unique to the *C. crescentus* lifestyle, such as adaptation to growing in oligotrophic environments and under different oxygen tensions. In fact, many TonB-dependent receptors, predicted to be required for sugar transport, and the hypoxia FixK regulon were surprisingly downregulated by both iron limitation and *fur* mutation.

### Verification of iron- and Fur-dependent expression of the CC2193 and CC3059 operons

Nearly all of the genes previously identified as members of the *C. crescentus* Fur regulon [6] were found to be differentially expressed by microarray analyses (Figure 2, red arrows), validating the experimental procedure. To further confirm our microarray data, we selected genes located in two clusters that encode putative transporters for validation by β-galactosidase activity assays and EMSA. The first cluster (CC2193-92-91) encodes a hypothetical protein containing an EF hand motif (CC2193), a putative glutathione peroxidase (CC2192) and a hypothetical protein (CC2191). The CC2193 gene appears to have been incorrectly annotated in the CB15 strain given that in the chromosome of the *C. crescentus* NA1000 strain, recently sequenced [43], two open reading frames were annotated in this region, CCNA02274 (encoding a shorter EF hand protein) and CCNA02275, encoding a hypothetical protein with a domain of unknown function (DUF4198). The second cluster (CC3059-60-61-62-63) contains three genes encoding a putative transporter (CC3059-60-61), and two genes involved in iron-related functions (sulfite reductase iron-flavoprotein and Fe-S cluster repair protein) (Figure 2; Table 1). Interestingly, the genes of these two clusters most highly upregulated in iron limitation and *fur* mutant (CC2193-corresponding to CCNA02275 in NA1000, and CC3061) (Table 1) encode two paralogous proteins belonging to the widespread Pfam family DUF4198. Although the proteins of this family are widely distributed in various groups of bacteria (750 sequences in 486 species, Pfam database February 2013), nothing is known about their function or regulation.

**Figure 3:**
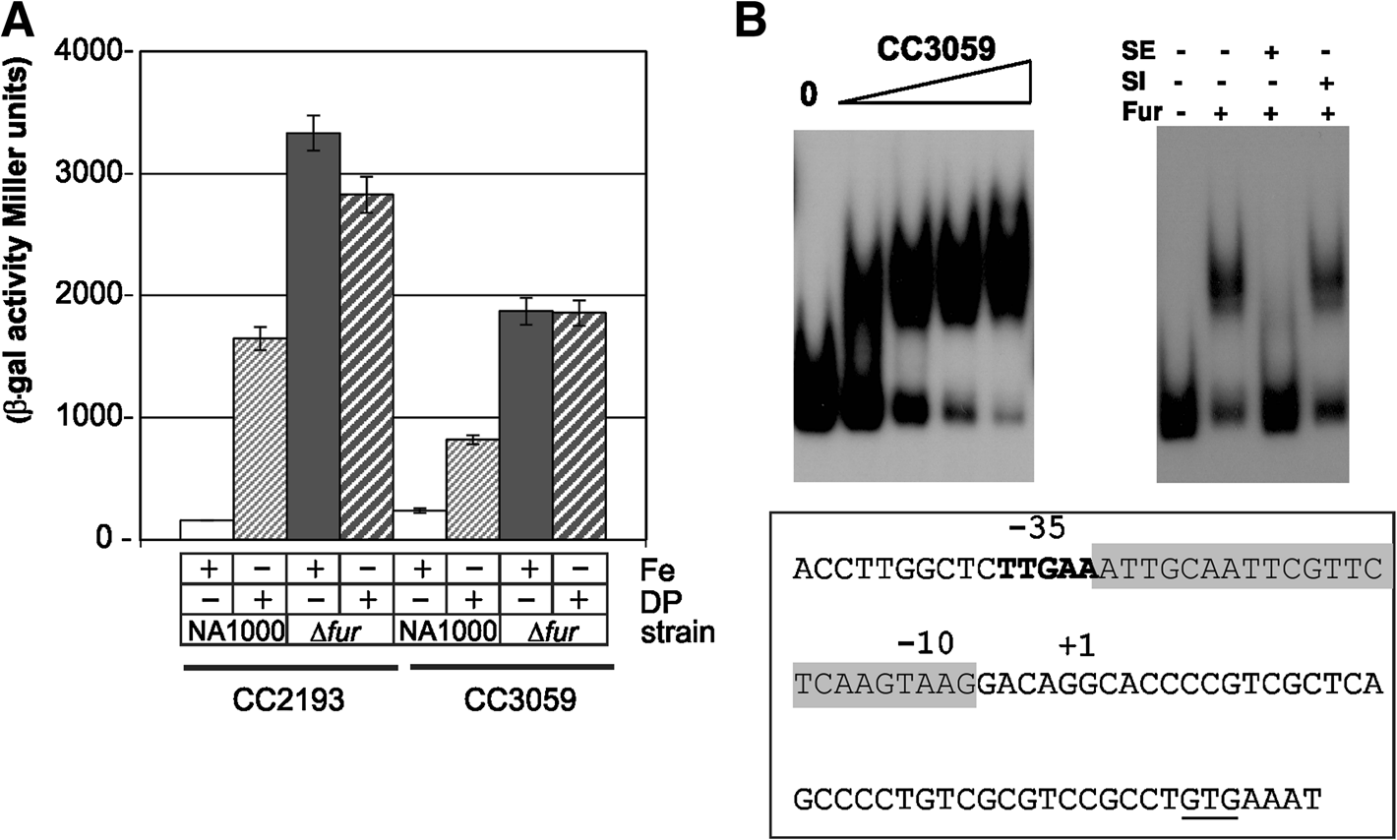
**The CC2193 and CC3059 operons are members of the *C. crescentus *Fur regulon. (A)** Promoter activities of the CC2193 and CC3059 operons in response to iron and Fur. Wild type (NA1000) and *fur* mutant (∆*fur*) strains containing plasmids pLAC2193 or pLAC3059 were grown in PYE medium and treated with 100 μM FeSO_4_ (Fe) or 100 μM 2,2-dipyridyl (DP) for two hours. The β-galactosidase activity generated by these *lacZ* fusions was determined. The experiments were performed in duplicate from three independent biological cultures. **(B)** Fur binds directly to the promoter of the CC3059 operon. EMSAs were performed using the purified His-Fur protein and a probe containing the promoter region of CC3059. The ^32^P-labeled probe was incubated with increasing concentrations of protein (0, 50, 200, 500 and 1000 nM) (left). A competition assay using 250 nM Fur and the labeled CC3059 probe was performed, where binding of Fur was challenged with a 30-fold excess of unlabeled DNA fragments of the same region (SE) or the 16S rRNA coding region (SI) as competitors (right). Below is shown the promoter region of the CC3059 operon, indicating the previously identified transcriptional start site (+1) and conserved −35 and −10 sequences of *Caulobacter* σ^70^ promoters (TTGAC-16 bp-G/CCTANA) [39]. The initiation codon (GTG) is underlined. The Fur binding site predicted *in silico* is shaded.

The promoter regions of CC2193 and CC3059 were cloned in a *lacZ* reporter plasmid and the constructions were introduced into the wild type and *fur* mutant strains. Beta-galactosidase activity assays indicated that the expression of these two genes was induced under iron limitation and derepressed in the *fur* mutant strain, validating the microarray data (Figure 3A, Table 1). To verify whether Fur acts as repressor by directly binding upstream of the CC2193-92-91 and CC3059-60-61-62-63 gene clusters (Figure 2) we performed gel mobility shift assays using purified Fur protein (Figure 3B). Fur bound to the probes corresponding to the promoter region of CC2193 (data not shown,[6]) as well as to that of CC3059, validating the Fur binding sites found overlapping the −35 and −10 promoter elements of these two genes (Figure 3B). These data illustrate the high performance of the microarray analysis to identify unknown genes with potential functions in iron acquisition and new members of the Fur regulon. For instance, the high derepression of CC2193 and CC3059 by iron limitation and *fur* mutation suggests that these two members of a widespread family of proteins with unknown function (DUF4198) could be involved in transport or signaling in response to iron limitation.

## Conclusions

**Figure 4:**
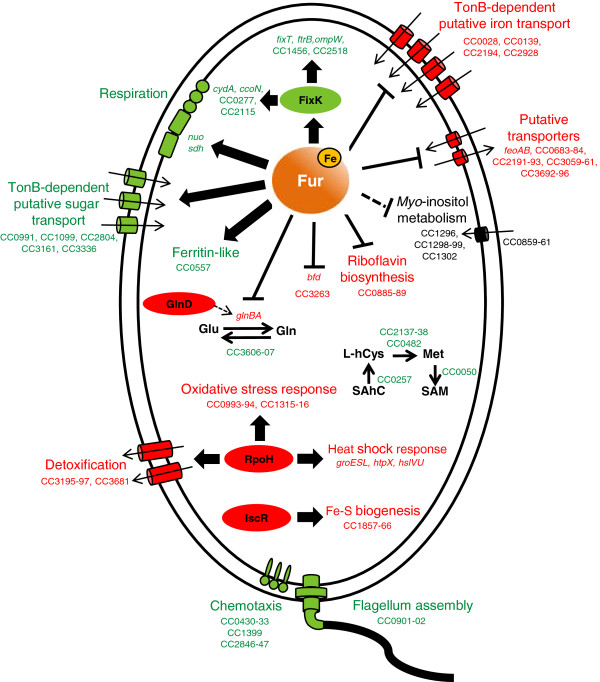
**Schematic representation of the main changes in gene expression and cell processes under iron-limiting conditions.** Upregulated genes and pathways are shown in red, downregulated are shown in green. Large arrows indicate activation and blunt-head lines indicate repression by the respective transcription regulator (Fur is represented as iron-bound). Thin arrows indicate enzyme reactions. Traced arrow indicates activation of the enzyme GlnB by GlnD via uridylylation. L-hCys: L-homocysteine, SAM: S-adenosylmethionine, SAhC: S-adenosylhomocysteine.

Using DNA microarray analyses, we have defined the global transcriptional response of *Caulobacter crescentus* to iron availability, providing an overview of the physiological strategies that this oligotrophic α-proteobacterium employs for survival in iron limiting conditions (Figure 4). Our data reveal that the iron stimulon in *C. crescentus* is larger than the Fur regulon previously identified [6], involving a more complex regulatory network. Among the responses mediated by Fur it is worth pointing out the upregulation of genes involved in iron acquisition systems and biosynthesis of riboflavin in iron limiting condition, as well as the downregulation of genes encoding many iron-using enzymes involved in energy metabolism (Figure 4). Fur binding site prediction suggests that Fur acts mainly as a direct transcriptional repressor, whereas positive regulation could be mediated either directly by Fur in a few cases or indirectly for most genes. In many cases this indirect effect was provoked by downregulation of the hypoxia regulator FixK, causing decreased expression of FixK-activated genes in iron limitation condition (Figure 4). Other genes could be indirectly activated by Fur via an unidentified iron-responsive sRNA. While the Fe^2+^-Fur mediated repression of some genes encoding TonB-dependent receptors confirmed our previous data [6], the Fe^2+^-Fur mediated activation of many other TonB-dependent receptors putatively associated with sugar transport was unexpected. Fur-independent regulation of *C. crescentus* iron-responsive genes was also observed, indicating an overlap with other regulatory pathways (Figure 4). It is worth mentioning that iron limitation caused upregulation of the heat shock sigma factor RpoH with consequent activation of its regulon, and upregulation of the IscR regulon, whose genes are involved in Fe-S cluster biogenesis. Since most of these iron-responsive genes identified in this work have not been experimentally investigated in *C. crescentus*, they are good targets for future studies.

## Methods

### Bacterial strains and growth conditions

*Caulobacter crescentus*, also known as *Caulobacter vibrioides*[44], strains NA1000 (wild-type) [45] and SP0057 (*fur* mutant) [6] were grown aerobically at 30°C in peptone-yeast extract (PYE) medium [46]. Iron-replete and iron-limiting conditions were achieved by supplementing PYE medium with 100 μM FeSO_4_ and 100 μM 2,2-dipyridyl (DP) (Sigma), respectively. Plasmids were introduced into *C. crescentus* by conjugation with *Escherichia coli* strain S17-1. *E. coli* was grown at 37°C in LB medium supplemented with ampicillin (100 μg ml^-1^) or tetracycline (12.5 μg ml^-1^) as required. His-Fur protein was purified after overexpression in *E. coli* DH5α as described [6].

### Microarray analysis

For the DNA microarray experiments, overnight *C. crescentus* cultures were diluted to an optical density at 600 nm (OD_600_) of 0.1 in 35 ml of PYE medium. Cells were grown up to midlog phase (OD_600_ ~ 0.5) and the cultures were divided and treated with either 100 μM FeSO_4_ (iron sufficiency) or 100 μM DP (iron limitation). The incubation was continued for two hours prior to RNA isolation as previously described [6]. Total RNA was extracted using Trizol Reagent (Invitrogen), according to the manufacturer’s instructions. RNA samples were treated with RNase-free DNase I (Fermentas) to digest residual chromosomal DNA and then precipitated using sodium acetate/ethanol prior to spectrophotometric quantification and visualization on formaldehyde-agarose gels. RNA samples were isolated from two independent bacterial cultures for each strain or condition analyzed as biological replicates. Amino allyl modified cDNA was generated by reverse transcription from 20 μg of total RNA and labeled with either Cy3 or Cy5 mono-reactive fluorescent dyes using the FairPlay III Microarray Labeling System (Stratagene). Labeled cDNA samples were hybridized to a custom-designed DNA oligo microarray (Agilent) (each gene is covered by 9–11 probes located −300 to +200 relative to the translational start site) using a protocol previously described [47,48]. The arrays were scanned for the Cy3 and Cy5 fluorescent signals with an Agilent High Resolution Microarray Scanner. Data extraction and normalization was performed with the Feature Extraction Software 9.0 (Agilent). A gene was considered as upregulated or downregulated if it showed 2-fold change relative to the control considering at least three out of four last probes (that are downstream of the translational start site) in both biological replicates. The values for the relative expression of each gene were obtained as the average of the four last probes. The microarray data have been deposited in the Gene Expression Omnibus (GEO) database (http://www.ncbi.nlm.nih.gov/geo) under accession number GSE45653.

### Fur binding site detection

Bioinformatics analyses were performed using the Multiple Em for Motif Elicitation (MEME) tool [49] to identify motifs within the promoter regions of iron regulated genes. Putative gene regulatory regions (−200 to +50 bp relative to the start codon) were searched using the following parameters: motifs size from 6 to 50 bp; zero or one motif per sequence; search given strand only; palindromic and nonpalindromic models were tested. Sequence logos were generated using WebLogo [50].

### *lacZ* fusions and ß-Galactosidase assays

DNA fragments covering the promoter regions of CC2193 (193 bp) and CC3059 (183 bp) were PCR-amplified using primer pairs CC2193-fw (5'-TGGATCCCGGCGAGTTTCAGGCGCGAC-3')/CC2193-rv (5'-TAAGCTTACGGATCATTGGACAAACCC-3') and CC3059-fw (5'-TGGATCCAGTTGACGGCGCAATAGGCC-3')/CC3059-rv (5'-TAAGCTTGCGGCGGCGGATTTCACAGG-3'), respectively. These PCR products were cloned into pGEM-T Easy, sequenced and subcloned as *BamH*I/*Hind*III fragments into the reporter vector pRK*lacZ*290 [51], resulting in plasmids pLAC2193 and pLAC3059. These constructs were introduced into *C. crescentus* NA1000 and SP0057 strains by conjugation. Cultures were grown in PYE medium up to mid-log phase, divided into two flasks, and treated with either 100 μM FeSO_4_ or 100 μM DP for two hours. The *ß*-galactosidase activity from these strains was determined colorimetrically using o-nitrophenyl-ß-D-galactoside (ONPG) as substrate [52].

### Electrophoretic mobility shift assay (EMSA)

A probe corresponding the promoter region of CC3059 (the same 183 bp- fragment used in *lacZ* fusion) was obtained by PCR amplification and was end-labeled with [γ^32^P]-ATP using T4 polynucleotide kinase (Invitrogen). For competition assay, a 101-bp 16S rRNA intragenic fragment was PCR-amplified using the primers 16SA-fw (5'-CCGCGTGAATGATGAAGGTC-3') and 16SA-rv (5'-GCTGCTGGCACGAAGTTAGC-3'). For EMSA, purified His-Fur protein and labeled DNA probes were incubated in binding buffer exactly as previously described [6].

## Competing interest

The authors declare that they have no competing interest.

## Authors’ contributions

JFSN and MVM planned the experiments; JFSN performed the experimental work and wrote the manuscript; JFSN and RFL analyzed the microarray data; MVM participated in study design and coordination and helped to prepare the manuscript. All authors read and approved the final manuscript.

## Supplementary Material

Additional file 1: Table S1 Differentially expressed genes in the *fur* mutant but not affected by iron limitation. Click here for file

## References

[B1] Andrews SC Robinson AK Rodriguez-Quinones F Bacterial iron homeostasis FEMS Microbiol Rev 2003 27 215 237 10.1016/S0168-6445(03)00055-X 12829269

[B2] Schaible UE Kaufmann SHE Iron and microbial infection Nat Rev Microbiol 2004 2 946 953 10.1038/nrmicro1046 15550940

[B3] Imlay JA Cellular defenses against superoxide and hydrogen peroxide Annu Rev Biochem 2008 77 755 776 10.1146/annurev.biochem.77.061606.161055 18173371 PMC3057177

[B4] Lee JW Helmann JD Functional specialization within the Fur family of metalloregulators Biometals 2007 20 485 499 10.1007/s10534-006-9070-7 17216355

[B5] Delany I Rappuoli R Scarlato V Fur functions as an activator and as a repressor of putative virulence genes in *Neisseria meningitidis* Mol Microbiol 2004 52 1081 1090 10.1111/j.1365-2958.2004.04030.x 15130126

[B6] da Silva Neto JF Braz VS Italiani VCS Marques MV Fur controls iron homeostasis and oxidative stress defense in the oligotrophic alpha-proteobacterium *Caulobacter crescentus* Nucleic Acids Res 2009 37 4812 4825 10.1093/nar/gkp509 19520766 PMC2724300

[B7] Yu C Genco CA Fur-mediated activation of gene transcription in the human pathogen *Neisseria gonorrhoeae* J Bacteriol 2012 194 1730 1742 10.1128/JB.06176-11 22287521 PMC3302472

[B8] Carpenter BM Whitmire JM Merrell DS This is not your mother's repressor: the complex role of Fur in pathogenesis Infect Immun 2009 77 2590 2601 10.1128/IAI.00116-09 19364842 PMC2708581

[B9] Butcher J Sarvan S Brunzelle JS Couture JF Stintzi A Structure and regulon of *Campylobacter jejuni* ferric uptake regulator Fur define apo-Fur regulation Proc Natl Acad Sci USA 2012 109 10047 10052 10.1073/pnas.1118321109 22665794 PMC3382491

[B10] Masse E Gottesman S A small RNA regulates the expression of genes involved in iron metabolism in *Escherichia coli* Proc Natl Acad Sci USA 2002 99 4620 4625 10.1073/pnas.032066599 11917098 PMC123697

[B11] Wilderman PJ Sowa NA FitzGerald DJ FitzGerald PC Gottesman S Ochsner UA Vasil ML Identification of tandem duplicate regulatory small RNAs in *Pseudomonas aeruginosa* involved in iron homeostasis Proc Natl Acad Sci USA 2004 101 9792 9797 10.1073/pnas.0403423101 15210934 PMC470753

[B12] Mellin JR Goswami S Grogan S Tjaden B Genco CA A novel Fur- and iron-regulated small RNA, NrrF, is required for indirect Fur-mediated regulation of the *sdhA* and *sdhC* genes in *Neisseria meningitidis* J Bacteriol 2007 189 3686 3694 10.1128/JB.01890-06 17351036 PMC1913314

[B13] Gaballa A Antelmann H Aguilar C Khakh SK Song KB Smaldone GT Helmann JD The *Bacillus subtilis* iron-sparing response is mediated by a Fur-regulated small RNA and three small, basic proteins Proc Natl Acad Sci USA 2008 105 11927 11932 10.1073/pnas.0711752105 18697947 PMC2575260

[B14] Masse E Salvail H Desnoyers G Arguin M Small RNAs controlling iron metabolism Curr Opin Microbiol 2007 10 140 145 10.1016/j.mib.2007.03.013 17383226

[B15] Rudolph G Hennecke H Fischer HM Beyond the Fur paradigm: iron-controlled gene expression in rhizobia FEMS Microbiol Rev 2006 30 631 648 10.1111/j.1574-6976.2006.00030.x 16774589

[B16] Johnston AW Todd JD Curson AR Lei S Nikolaidou-Katsaridou N Gelfand MS Rodionov DA Living without Fur: the subtlety and complexity of iron-responsive gene regulation in the symbiotic bacterium *Rhizobium* and other alpha-proteobacteria Biometals 2007 20 501 511 10.1007/s10534-007-9085-8 17310401

[B17] Rodionov DA Gelfand MS Todd JD Curson AR Johnston AW Computational reconstruction of iron- and manganese-responsive transcriptional networks in alpha-proteobacteria PLoS Comput Biol 2006 2 e163 10.1371/journal.pcbi.0020163 17173478 PMC1698941

[B18] Uebe R Voigt B Schweder T Albrecht D Katzmann E Lang C Böttger L Matzanke B Schüler D Deletion of a *fur*-like gene affects iron homeostasis and magnetosome formation in *Magnetospirillum gryphiswaldense* J Bacteriol 2010 192 4192 4204 10.1128/JB.00319-10 20562310 PMC2916424

[B19] Qi L Li J Zhang W Liu J Rong C Li Y Wu L Fur in *Magnetospirillum gryphiswaldense* influences magnetosomes formation and directly regulates the genes involved in iron and oxygen metabolism PLoS ONE 2012 7 e29572 10.1371/journal.pone.0029572 22238623 PMC3251581

[B20] Boutte CC Srinivasan BS Flannick JA Novak AF Martens AT Batzoglou S Viollier PH Crosson S Genetic and computational identification of a conserved bacterial metabolic module PLoS Genet 2008 4 e1000310 10.1371/journal.pgen.1000310 19096521 PMC2597717

[B21] McHugh JP Rodriguez-Quinones F Abdul-Tehrani H Svistunenko DA Poole RK Cooper CE Andrews SC Global iron-dependent gene regulation in *Escherichia coli*. A new mechanism for iron homeostasis J Biol Chem 2003 278 29478 29486 10.1074/jbc.M303381200 12746439

[B22] Christen B Abeliuk E Collier JM Kalogeraki VS Passarelli B Coller JA Fero MJ McAdams HH Shapiro L The essential genome of a bacterium Mol Syst Biol 2011 7 528 21878915 10.1038/msb.2011.58 PMC3202797

[B23] Worst DJ Gerrits MM Vandenbrouke-Grauls CM Kusters JG *Helicobacter pylori ribBA*-mediated riboflavin production is involved in iron acquisition J Bacteriol 1998 180 1473 1479 9515916 10.1128/jb.180.6.1473-1479.1998 PMC107047

[B24] Crossley RA Gaskin DJ Holmes K Mulholland F Wells JM Kelly DJ van Vliet AH Walton NJ Riboflavin biosynthesis is associated with assimilatory ferric reduction and iron acquisition by *Campylobacter jejuni* Appl Environ Microbiol 2007 73 7819 7825 10.1128/AEM.01919-07 17965203 PMC2168145

[B25] López-Gomollón S Hernández JA Pellicer S Angarica VE Peleato ML Fillat MF Cross-talk between iron and nitrogen regulatory networks in *Anabaena (Nostoc) sp*. PCC 7120: identification of overlapping genes in FurA and NtcA regulons J Mol Biol 2007 374 267 281 10.1016/j.jmb.2007.09.010 17920076

[B26] Landt SG Abeliuk E McGrath PT Lesley JA McAdams HH Shapiro L Small non-coding RNAs in *Caulobacter crescentus* Mol Microbiol 2008 68 600 614 10.1111/j.1365-2958.2008.06172.x 18373523 PMC7540941

[B27] Masse E Vanderpool CK Gottesman S Effect of RyhB small RNA on global iron use in *Escherichia coli* J Bacteriol 2005 187 6962 6971 10.1128/JB.187.20.6962-6971.2005 16199566 PMC1251601

[B28] Baichoo N Wang T Ye R Helmann JD Global analysis of the *Bacillus subtilis* Fur regulon and the iron starvation stimulon Mol Microbiol 2002 45 1613 1629 10.1046/j.1365-2958.2002.03113.x 12354229

[B29] England JC Perchuk BS Laub MT Gober JW Global regulation of gene expression and cell differentiation in *Caulobacter crescentus* in response to nutrient availability J Bacteriol 2010 192 819 833 10.1128/JB.01240-09 19948804 PMC2812448

[B30] Landt SG Lesley JA Britos L Shapiro L CrfA, a small noncoding RNA regulator of adaptation to carbon starvation in *Caulobacter crescentus* J Bacteriol 2010 192 4763 4775 10.1128/JB.00343-10 20601471 PMC2937403

[B31] Schauer K Rodionov DA de Reuse H New substrates for TonB-dependent transport: do we only see the ‘tip of the iceberg’? Trends Bioch Sci 2008 33 330 338 10.1016/j.tibs.2008.04.012 18539464

[B32] Crosson S McGrath PT Stephens C McAdams HH Shapiro L Conserved modular design of an oxygen sensory/signaling network with species-specific output Proc Natl Acad Sci USA 2005 102 8018 8023 10.1073/pnas.0503022102 15911751 PMC1142393

[B33] Troxell B Fink RC Porwollik S McClelland M Hassan HM The Fur regulon in anaerobically grown *Salmonella enterica sv* Typhimurium: identification of new Fur targets. BMC Microbiol 2011 11 236 10.1186/1471-2180-11-236 PMC3212961 22017966

[B34] Outten FW Djaman O Storz G A *suf* operon requirement for Fe-S cluster assembly during iron starvation in *Escherichia coli* Mol Microbiol 2004 52 861 872 10.1111/j.1365-2958.2004.04025.x 15101990

[B35] Py B Barras F Building Fe-S proteins: bacterial strategies Nat Rev Microbiol 2010 8 436 446 10.1038/nrmicro2356 20467446

[B36] Zheng M Wang X Templeton LJ Smulski DR LaRossa RA Storz G DNA microarray-mediated transcriptional profiling of the *Escherichia coli* response to hydrogen peroxide J Bacteriol 2001 183 4562 4570 10.1128/JB.183.15.4562-4570.2001 11443091 PMC95351

[B37] Hu P Brodie EL Suzuki Y McAdams HH Andersen GL Whole-genome transcriptional analysis of heavy metal stresses in *Caulobacter crescentus* J Bacteriol 2005 187 8437 8449 10.1128/JB.187.24.8437-8449.2005 16321948 PMC1317002

[B38] Yang Y Harris DP Luo F Xiong W Joachimiak M Wu L Dehal P Jacobsen J Yang Z Palumbo AV Arkin AP Zhou J Snapshot of iron response in *Shewanella oneidensis* by gene network reconstruction BMC Genomics 2009 10 131 10.1186/1471-2164-10-131 19321007 PMC2667191

[B39] McGrath PT Lee H Zhang L Iniesta AA Hottes AK Tan MH Hillson NJ Hu P Shapiro L McAdams HH High-throughput identification of transcription start sites, conserved promoter motifs and predicted regulons Nat Biotechnol 2007 25 584 592 10.1038/nbt1294 17401361

[B40] Wu J Newton A The *Caulobacter* heat shock sigma factor gene *rpoH* is positively autoregulated from a sigma32-dependent promoter J Bacteriol 1997 179 514 521 8990305 10.1128/jb.179.2.514-521.1997 PMC178723

[B41] Chao TC Buhrmester J Hansmeier N Pühler A Weidner S Role of the regulatory gene *rirA* in the transcriptional response of *Sinorhizobium meliloti* to iron limitation Appl Environ Microbiol 2005 71 5969 5982 10.1128/AEM.71.10.5969-5982.2005 16204511 PMC1265945

[B42] Eijkelkamp BA Hassan KA Paulsen IT Brown MH Investigation of the human pathogen *Acinetobacter baumannii* under iron limiting conditions BMC Genomics 2011 12 126 10.1186/1471-2164-12-126 21342532 PMC3055841

[B43] Marks ME Castro-Rojas CM Teiling C Du L Kapatral V Walunas TL Crosson S The genetic basis of laboratory adaptation in *Caulobacter crescentus* J Bacteriol 2010 192 3678 3688 10.1128/JB.00255-10 20472802 PMC2897358

[B44] Abraham WR Strömpl C Meyer H Lindholst S Moore ER Christ R Vancanneyt M Tindall BJ Bennasar A Smit J Tesar M Phylogeny and polyphasic taxonomy of *Caulobacter* species. Proposal of *Maricaulis* gen. nov. with *Maricaulis maris* (Poindexter) comb. nov. as the type species, and emended description of the genera *Brevundimonas* and *Caulobacter* Int J Syst Bacteriol 1999 49 1053 1073 10.1099/00207713-49-3-1053 10425763

[B45] Evinger M Agabian N Envelope-associated nucleoid from *Caulobacter crescentus* stalked and swarmer cells J Bacteriol 1977 132 294 301 334726 10.1128/jb.132.1.294-301.1977 PMC221855

[B46] Ely B Genetics of Caulobacter crescentus. Methods Enzymol 1991 204 372 384 10.1016/0076-6879(91)04019-k 1658564

[B47] Lourenço RF Kohler C Gomes SL A two-component system, an anti-sigma factor and two paralogous ECF sigma factors are involved in the control of general stress response in *Caulobacter crescentus* Mol Microbiol 2011 80 1598 1612 10.1111/j.1365-2958.2011.07668.x 21564331

[B48] Kohler C Lourenço RF Avelar GM Gomes SL Extracytoplasmic function (ECF) sigma factor σ^F^ is involved in *Caulobacter crescentus* response to heavy metal stress BMC Microbiology 2012 12 210 10.1186/1471-2180-12-210 22985357 PMC3511200

[B49] Bailey TL Elkan C Fitting a mixture model by expectation maximization to discover motifs in biopolymers Proceedings of the Second International Conference on Intelligent Systems for Molecular Biology 1994 Menlo Park, CA: AAAI Press 28 36 7584402

[B50] Crooks GE Hon G Chandonia JM Brenner SE WebLogo: a sequence logo generator Genome Res 2004 14 1188 1190 10.1101/gr.849004 15173120 PMC419797

[B51] Gober JW Shapiro L A developmentally regulated *Caulobacter* flagellar promoter is activated by 3' enhancer and IHF binding elements Mol Biol Cell 1992 3 913 916 10.1091/mbc.3.8.913 1392079 PMC275648

[B52] Miller JH Experiments in Molecular Genetics 1972 Cold Spring Harbor, New York: Cold Spring Harbor Laboratory Press

