# Supplementary material for: Global transcriptional response of Caulobacter crescentus to iron availability
Source: BMC Genomics. 2013 Aug 13;14:549. doi: 10.1186/1471-2164-14-549 (PMC3751524; doi:10.1186/1471-2164-14-549)
Supplement: Additional file 1: Table S1 — Differentially expressed genes in the fur mutant but not affected by iron limitation. [file 1471-2164-14-549-S1.docx]

**Table S1** – **Differentially expressed genes in the *fur* mutant but not affected by iron limitation**

| **Gene CB15** | **Gene NA1000** | **Predicted Function** | **Fold change^a^** |
| --- | --- | --- | --- |
| **Upregulated** | | | |
| CC_0042 | CCNA_00041 | bacterial protein translation initiation factor 2 IF-2 | 2.46 |
| CC_0369 | CCNA_00374 | putative ATP synthase protein I | 2.76 |
| CC_0608 | CCNA_00644 | conserved hypothetical protein | 3.42 |
| CC_0611 | CCNA_00647 | nitrate transport ATP-binding protein nrtD | 2.55 |
| CC_0616 | CCNA_00652 | nitrite reductase (NAD(P)H) small subunit (Fe-S cluster) | 3.86 |
| CC_0835^b^ | CCNA_00878 | ATP-dependent RNA helicase | 2.36 |
| CC_0859 | CCNA_00902 | inositol ABC transporter, periplasmic inositol-binding protein IbpA | 5.46 |
| CC_0860 | CCNA_00903 | inositol transport ATP-binding protein IatA | 4.00 |
| CC_0861 | CCNA_00904 | inositol ABC transport system, permease | 3.01 |
| CC_1296 | CCNA_01354 | myo-inositol 2-dehydrogenase IdhA | 4.59 |
| CC_1298 | CCNA_01356 | 5-dehydro-2-deoxygluconokinase IolC | 4.98 |
| CC_1299 | CCNA_01357 | myo-inositol catabolism protein IolD | 4.54 |
| CC_1302 | CCNA_01360 | malonate-semialdehyde dehydrogenase IolA | 4.60 |
| CC_1339 | CCNA_01400 | nitrogen regulatory protein GlnK | 3.77 |
| CC_2388 | CCNA_02471 | cobalt-zinc-cadmium resistance protein czcC | 3.89 |
| CC_3297 | CCNA_03406 | SSU ribosomal protein S21P | 3.49 |
| **Downregulated** | | | |
| CC_0057 | CCNA_00055 | ferric uptake regulation protein | -7.46 |
| CC_0128 | CCNA_00127 | hypothetical protein | -3.38 |
| CC_0210^c^ | CCNA_00210 | TonB-dependent receptor | -3.00 |
| CC_0353 | CCNA_00358 | delta 3,5-delta, 4-dienoyl-CoA isomerase precursor | -2.62 |
| CC_0446 | CCNA_00455 | glucosamine/chitin transporter nagA (TonB-depedent receptor) | -2.83 |
| CC_0556^c^ | CCNA_00591 | hypothetical protein (predicted catalase) | -2.22 |
| CC_0557^c^ | CCNA_00592 | hypothetical protein (Ferritin-like) | -2.06 |
| CC_0762^c^ | CCNA_00800 | cytochrome bd-type quinol oxidase, subunit 1 cydA | -3.52 |
| CC_0944 | CCNA_00993 | long-chain-fatty-acid--CoA ligase | -2.64 |
| CC_1229 | CCNA_01287 | epoxide hydrolase | -2.19 |
| CC_1232^c^ | CCNA_01290 | hypothetical protein | -2.08 |
| CC_1233^c^ | CCNA_01291 | phosphotransferase family protein | -2.09 |
| CC_1396 | CCNA_01462 | lactate 2-monooxygenase | -2.13 |
| CC_1411 | CCNA_01477 | oxygen-independent coproporphyrinogen-III oxidase hemN | -2.66 |
| CC_1541^c^ | CCNA_01610 | 2-isopropylmalate synthase | -2.26 |
| CC_1628^c^ | CCNA_01700 | nucleoside permease (Major Facilitator Superfamily) | -2.38 |
| CC_1633 | CCNA_01705 | conserved hypothetical protein (DUF1080) | -2.28 |
| CC_1634 | CCNA_01706 | dehydrogluconate dehydrogenase (GMC oxidoreductase) | -2.45 |
| CC_1635 | CCNA_01707 | conserved hypothetical protein (Gluconate 2-dehydrogenase) | -2.40 |
| CC_1764 | CCNA_01841 | socitrate lyase | -3.80 |
| CC_1765 | CCNA_01843 | malate synthase | -2.38 |
| CC_1849 | CCNA_01925 | coniferyl aldehyde dehydrogenase | -2.80 |
| CC_1950^c^ | CCNA_02027 | NADH dehydrogenase subunit E (Fe-S cluster) | -2.03 |
| CC_1955^c^ | CCNA_02032 | NADH dehydrogenase subunit B (Fe-S cluster) | -2.05 |
| CC_1956^c^ | CCNA_02033 | NADH dehydrogenase subunit A | -2.00 |
| CC_2642 | CCNA_02725 | choline dehydrogenase (GMC oxireductase) | -2.18 |
| CC_2823 | CCNA_02914 | zinc metalloprotease | -2.10 |
| CC_2824 | CCNA_02915 | microcin-processing peptidase 1 (PmbA) | -2.70 |
| CC_2970 | CCNA_03065 | cytochrome c family protein | -2.52 |
| CC_3088^c^ | CCNA_03185 | glutathione S-transferase | -2.23 |
| CC_3189^c^ | CCNA_03293 | enoyl-CoA hydratase | -2.09 |
| CC_3207^c^ | CCNA_03313 | hypothetical protein | -3.18 |
| CC_3338 | CCNA_03446 | feruloyl-CoA synthetase | -2.74 |
| CC_3459 | CCNA_03572 | putative cytosolic protein (DUF1330) | -2.15 |
| CC_3466 | CCNA_03579 | hypothetical protein (DUF883) | -3.37 |
| CC_3557 | CCNA_03672 | iron/manganese superoxide dismutase | -2.21 |

^a^ Values are fold change in the expression levels comparing *fur* mutant strain *versus* wild type strain both exposed to iron-replete condition (∆fur Fe/WT Fe).

^b^This gene is probably also upregulated in iron limitation since its expression change was very close to our cutoff criterion.

^c^These genes are probably also downregulated in iron limitation since their expression changes were very close to our cutoff criterion.
